# Efficient Conversion of Glucose into Lactic Acid over the Lewis Acidity Enhanced Sn-Beta Catalyst

**DOI:** 10.3390/molecules30071457

**Published:** 2025-03-25

**Authors:** Fenfen Guo, Yuxuan Wang, Zhicheng Jiang, Youjing Tu, Ruikai Li, Xingyu Zhang, Aoyi Tang, Yuan Liang, Lishi Yan, Hu Luo, Shenggang Li, Lingzhao Kong

**Affiliations:** 1 School of Environmental Science and Engineering, Suzhou University of Science and Technology, Suzhou 215009, China; 2213022037@post.usts.edu.cn (F.G.); 2213022044@post.usts.edu.cn (Y.W.); 2213022010@post.usts.edu.cn (Z.J.); 2313021012@post.usts.edu.cn (Y.T.); 2313021004@post.usts.edu.cn (R.L.); 2313022025@post.usts.edu.cn (X.Z.); 2313021008@post.usts.edu.cn (A.T.); liangyuan@mail.usts.edu.cn (Y.L.); 2 School of Chemistry and Life Sciences, Suzhou University of Science and Technology, Suzhou 215009, China; yanls@usts.edu.cn; 3 CAS Key Laboratory of Low-Carbon Conversion Science and Engineering, Shanghai Advanced Research Institute, Chinese Academy of Sciences, Shanghai 201210, China; lisg@sari.ac.cn

**Keywords:** Sn-Beta catalyst, glucose, lactic acid, Lewis acidity

## Abstract

The catalytic production of lactic acid from carbohydrates was considered a green way to efficiently utilize renewable biomass resources. In this study, an easy post-synthesis method was used to prepare a Sn-Beta catalyst for the production of lactic acid from glucose at 180 °C, 2 MPa, and 30 min. With optimized reaction time, temperature, pressure, and the ratio of raw material to catalyst, the yield of lactic acid reached an astonishingly high level of 76.0%. In addition, the catalyst characterizations were performed in-depth, revealing the intrinsic relationship between catalyst performance and structure, proving that the 2 wt% Sn was uniformly dispersed in the skeleton of Beta zeolite, which significantly increased the density of Lewis acid. Thus, the enhanced isomerization and retro-aldol condensation processes over the Lewis acid sites led to the high yield of lactic acid. This catalytic system kept stable after five cycles at mild conditions, showing high potential for industrial biomass utilization.

## 1. Introduction

Biomass, as the only renewable carbon source on the earth [1], is abundant and widely distributed in nature [2]. Biomass is an effective means to achieve carbon neutrality, and its conversion process can achieve near-zero carbon emissions [3]. In the context of circular economy and sustainable development, the efficient utilization of biomass resources for the preparation of chemicals and fuels is one of the most effective means to address the rapid depletion of fossil energy sources, climate change consequences, and prominent environmental issue [4,5,6]. Among them, the preparation of high value chemicals, especially oxygenated chemicals [7,8,9] (e.g., lactic acid [10], levulinic acid [11], 5-hydroxymethyl furfural [12,13]) from biomass by homogeneous and heterogenous catalysts are very attractive directions. Lactic acid served as an important platform chemical compound, especially as the structural monomer of the biodegradable plasticizer polylactic acid (PLA) [14]; the increase in demand has driven the large-scale production of lactic acid. Currently, microbial fermentation is the primary method for lactic acid production [15]. Nevertheless, there are notable disadvantages in the fermentation production process, including the elevated costs of raw materials and the rigorous criteria for strain selection. Additionally, the production cycle tends to be lengthy, and these elements can significantly affect overall production efficiency and economic feasibility [16]. In comparison, chemical catalysis holds significant potential in the development and utilization of biomass for the production of various chemicals due to its rapid reaction time and high efficiency. Researchers have explored homogeneous and heterogeneous catalyst systems for the conversion of biomass into lactic acid [17]. Usually, for easy separation, heterogeneous catalysts such as solid acids and bases, zeolites, and metal oxides were used for preparing lactic acid from bio-based feedstocks [18]. Among the known catalysts, Lewis acid sites produced by isolated Sn (IV) in Sn-Beta zeolites enable it to exhibit excellent catalytic performance in carbohydrate isomerization and retro-aldolization reactions [19,20]. In 2010, Holm et al. [21] investigated the conversion of sucrose to lactic acid (or its ester) in water/methanol using Sn-Beta as a Lewis acid catalyst, resulting in a yield of 28.0%/68.0%. To further enhance the yield of lactic acid, especially in the aqueous phase, further works on modified catalysts and the introduction of solvents or an external field were reported. Sun et al. [22] synthesized template-free Sn-Beta zeolite through an oil-heating crystallization method, resulting in a lactic acid yield of 67.1% within 30 min at 200 °C and 4.0 MPa. Zhang et al. [23] reported that the yield of lactic acid from xylose (C5 sugar) using Sn-Beta catalyst could reach 70.0%. Zhao et al. [24] obtained a 72% yield of lactic acid over the post-synthesized Sn-Beta catalyst by employing 2.0 wt% γ-valerolactone (GVL) as an additive in water. Liu et al. [25] introduced microwaves into the isomerization of glucose to promote its effective catalytic conversion over a “monolithic” Sn-Beta catalyst in water, and the reaction was carried out at 180 °C for 30 min, leading to a high yield of lactic acid of 68.3%. The above-mentioned works revealed that the catalytic performance largely depends on the effective tuning of the acid–base equilibrium of the Sn-Beta catalyst. Therefore, how to further modulate the catalyst so that the reaction can be carried out at an even milder environment with higher activity and product selectivity is of great significance and highly attractive.

In this work, we used a commercial Beta zeolite with SiO_2_:Al_2_O_3_ = 100 instead of the original Beta with SiO_2_:Al_2_O_3_ = 25 to prepare the Sn-Beta with a 2 wt% Sn loading by post-synthesis. The yield of lactic acid reached the higher value of 76.0% at the lower reaction temperature of 180 °C for 30 min. A series of characterizations was carried out for the Sn-Beta catalyst to explore the effect of the acid–base equilibrium on the catalytic performance.

## 2. Results and Discussion

### 2.1. Catalytic Performance of Sn-Beta for Glucose Conversion

**Figure 1:**
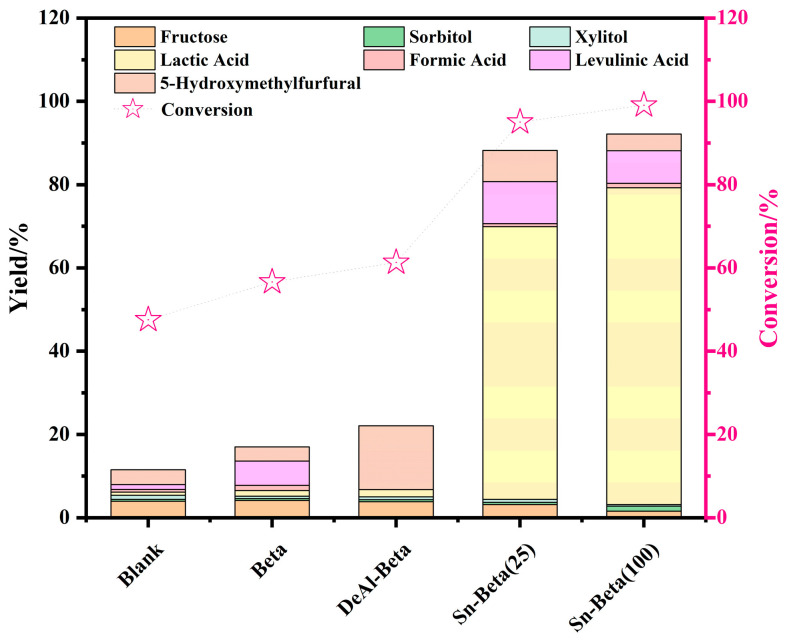
Conversion rates of glucose and yield of lactic acid over different studied catalysts. Reaction conditions: 200.0 mg glucose, 20.0 mL deionized water, 200.0 mg catalyst, 2 MPa N_2_, 180 °C, 30 min.

Firstly, we performed controlled experiments with glucose as the substrate using different catalysts, as shown in Figure 1. In the absence of catalyst loading, the variety of products is relatively limited, and the yields of most products are low, thereby constraining both the conversion rate of the reaction and the diversity of the products. When the commercial Beta zeolite was used as the catalyst, only 1.3% of lactic acid, along with 3.4% of 5-hydroxymethyl furfural (HMF), 7.8% of levulinic acid, and 1.08% of formic acid were produced, which could be attributed to the fact that the Brønsted acid sites in the Beta zeolite promoted the dehydration of glucose to produce HMF and insoluble humins. When using the de-aluminized Beta as the catalyst, 1.7% of lactic acid and 15.3% of HMF were obtained, which was due to the relatively strong Brønsted acidity shown by the rich silica hydroxyl defective sites on the de-Al Beta. Surprisingly, the yield of lactic acid reached 76.0% when using the Sn-Beta catalyst prepared from the commercial Beta zeolite with SiO_2_:Al_2_O_3_ = 100 under the optimal conditions of 180 °C and 2 MPa for 30 min (Figures S1–S4). Furthermore, the yield of lactic acid was also enhanced by about 10.5% compared to a similar catalyst using Beta zeolite with SiO_2_:Al_2_O_3_ = 25 (65.5%). Moreover, the catalytic activity of the Sn-Beta remained relatively stable after five regeneration cycles (Figure S7). The ICP results (Table S1) showed that the Sn content was essentially the same as the initial content. High lactic acid yields were maintained with only minor losses of 8.1% (Figure S8) in the reaction. The carbon equilibrium in the liquid phase is nearly close to 100%.

### 2.2. Characterization of Sn-Beta Catalyst

**Figure 2:**
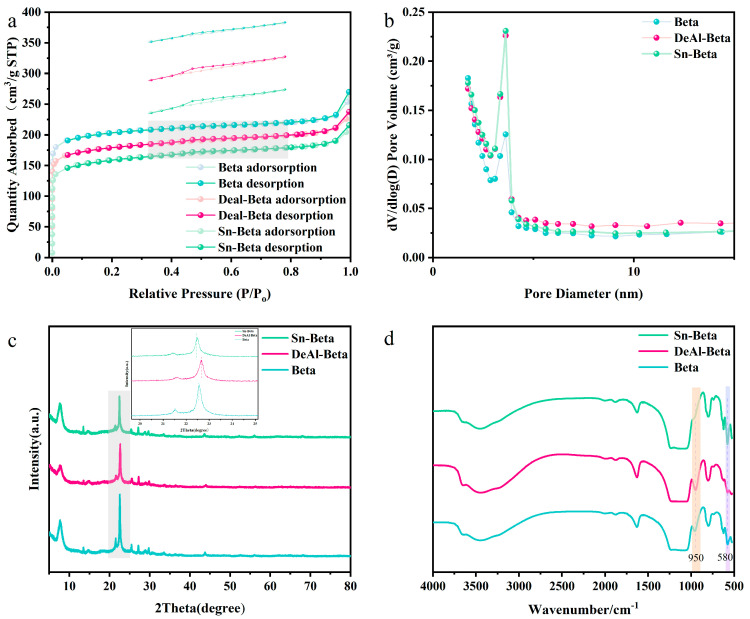
(**a**) N_2_ adsorption-desorption isotherms. (**b**) The corresponding mesopore size distributions of the samples. (**c**) XRD patterns. (**d**) FTIR spectra of Beta, deAl-Beta and Sn-Beta.

The SEM images (Figure S5a) showed that the synthesized Sn-Beta catalyst has a clean surface and consists of regularly shaped microsphere-type nanoparticles with grain sizes around 100 nm, which were relatively small in size and thus favored the diffusion of reactants and products. In addition, TEM-mapping images (Figure S5b) of the Sn-Beta catalyst reveal that the Sn species are uniformly distributed in Sn-Beta. Figure 2a,b show the N_2_ adsorption and desorption and the pore size distribution curves of Beta, deAl-Beta, and Sn-Beta, where all the curves displayed the type IV adsorption [26], indicating that all the materials have a mesoporous structure. As summarized in Table S1, the physicochemical characteristics of Beta, Deal-Beta, and Sn-Beta samples demonstrate that de-alumination leads to moderate decreases in both specific surface area and micropore volume. Notably, subsequent tin incorporation effectively restores these parameters, with Sn-Beta exhibiting comparable pore volume values to the parent Beta material, indicating that the tin modification protocol preserves the structural integrity of the zeolite framework without compromising its crystallographic architecture [27,28].

XRD patterns of the Beta, deAl-Beta and Sn-Beta catalysts are shown in Figure 2c. These patterns have strong characteristic peaks at 2θ = 7.8°, 21.5° and 22.5°, which are the typical BEA topological peaks of Beta crystals, indicating that the treatment by HNO_3_ and the addition of Sn did not affect the framework structure of the Beta catalyst [29]. No characteristic peaks of SnO_2_ were detected in the XRD patterns, indicating that most of the Sn species were dispersed into the zeolite skeleton. Furthermore, the intensities of the deAl-Beta and Sn-Beta peaks were somewhat reduced compared with the Beta zeolite, suggesting that de-alumination will cause some damage to the zeolite surface, although its crystal structure will be retained [30]. A comparison of the 2θ angles of the parent Beta (22.5°), the deAl-Beta (22.6°), and the Sn-Beta (22.4°) revealed contraction and dilation of the zeolite lattice caused by the removal of Al atoms and the successful incorporation of Sn species into the zeolite skeleton [31]. From the FTIR analysis, the peak at 580 cm^−1^ was the characteristic vibration of the Beta zeolite structure (Figure 2d) [32]. Compared to Beta, deAl-Beta showed a distinct peak around 950 cm^−1^, which is due to silanol defects from Al removal. The peak disappeared when Sn was doped, suggesting the formation of a four-coordinated skeletal Sn species [33], which is in agreement with XRD results.

**Figure 3:**
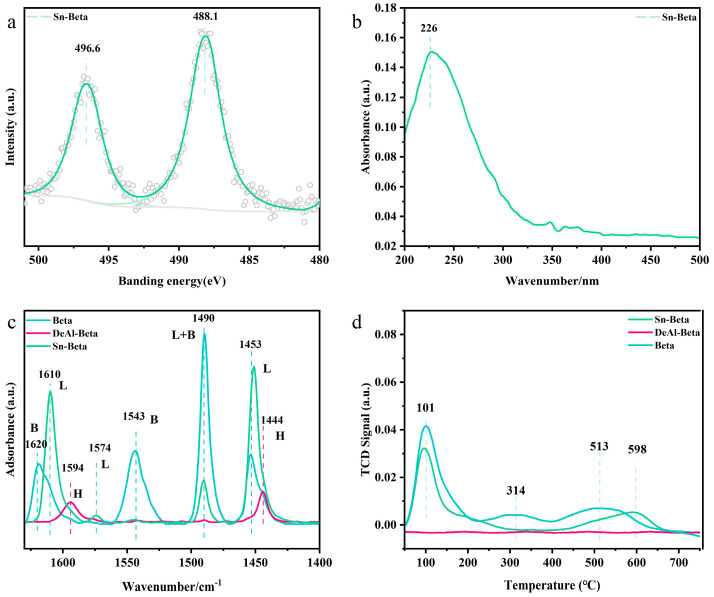
(**a**) XPS, (**b**) UV–vis of Sn–Beta; (**c**) Py–IR of Beta deAl–Beta and Sn–Beta (L: Lewis cid; B: Brønsted; H: hydrogen bonding); (**d**) NH_3_–TPD of Beta and Sn–Beta.

In the XPS analysis of the Sn-Beta catalyst, the spectrum (Figure 3a) reveals a doublet located at 488.1 eV and 496.6 eV [34]. According to published studies, these peaks can be attributed to the 3d5/2 and 3d3/2 electrons of tetrahedrally coordinated Sn^4+^ ions. Notably, these signals differ from those of pure SnO_2_, where the 3d5/2 and 3d3/2 orbitals typically appear at 486.0 eV and 494.5 eV, respectively [24]. This discrepancy indicates that the Sn species have been successfully incorporated into the framework structure of the Sn-Beta zeolite in tetrahedral coordination rather than existing as isolated SnO_2_ entities.

As shown in Figure 3b, the UV–vis results showed a distinct absorption peak at 210–230 nm attributed to charge transfer between O and isolated tetrahedral Sn^4+^, indicating that Sn was successfully embedded into the backbone of the form of tetra-coordinated Sn, consistent with the XPS results [35].

For the Py-IR spectra of the Beta zeolite, the peaks at 1543 cm^−1^ and 1620 cm^−1^ were due to Brønsted acid sites produced by bridging Al-OH-Si [36], and the absorption at 1490 cm^−1^ was attributed to the adsorption of pyridine on the Brønsted and Lewis acid sites [37] (Figure 3c). For de-Al-Beta, the absorption peaks at 1444 cm^−1^ and 1594 cm^−1^ were attributed to the hydrogen bonding interaction between the surface silanol and the pyridine, suggesting that abundant silanol nests are produced after complete de-alumination resulting in a decrease in acidity. The absorption peaks at 1453 cm^−1^ and 1610 cm^−1^ were due to the binding of pyridine to the strong Lewis acid site, whereas that at 1574 cm^−1^ was the binding of the pyridine to the Lewis acid sites [38], indicating the presence of strong and weak Lewis acids in Sn-Beta catalyst. The absorption peak at 1453 cm^−1^ reappeared after the addition of Sn, which resulted in a strong Lewis acid site, and the decrease in the peak at 1594 cm^−1^ was due to the entry of Sn species into the skeleton to restore the structure of the molecular sieves [39]. The reduced peak at 1490 cm^−1^ indicated that the Sn-Beta catalyst exhibited weak Brønsted acid sites. The amount of Lewis acid and Brønsted acid sites were shown in Table S3, and modifying the Sn-Beta catalyst significantly increased the density of its Lewis acid, decreased the density of its Brønsted acid, and lowered the ratio of Brønsted/Lewis acid sites, thereby inhibiting the formation of by-products and facilitating the production of lactic acid. NH_3_-TPD (Figure 3d) confirmed that both Beta and Sn-Beta consisted of weak (101 °C) and strong acid sites (513 °C and 598 °C), and the difference lies in that the HNO_3_ treatment and the addition of Sn reduce the medium-strength acid sites (314 °C) and decrease the amount of acid (as shown in Table S4) [40]. The absence of significant desorption peaks in the de-aluminized zeolite indicated that there are no strong or weak acid sites in the deAl-Beta zeolite, suggesting that the de-aluminization process has been successful [41]. Therefore, the appropriate acid strength and the ratio of Lewis/Brønsted acid sites are critical for the efficient production of lactic acid from glucose [42].

**Scheme 1:**
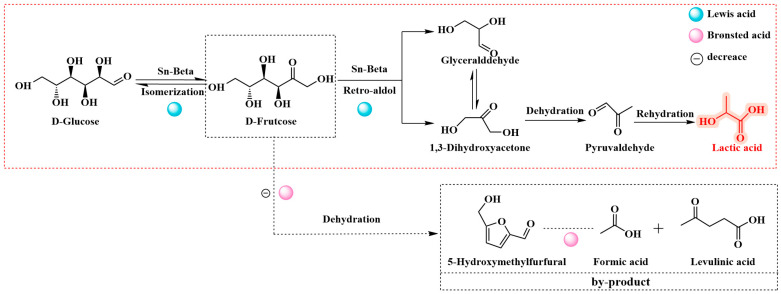
Proposed reaction pathway for the convention of glucose to lactic acid.

The reaction pathway of lactic acid formation can be usually divided into three stages: the initial glucose isomerization to fructose, the subsequent inverse hydroxyl aldol condensation fructose to produce the C3 intermediates 1,3-dihydroxyacetone and glycerolaldehyde, which can be interconverted by isomerization, and then further dehydrated and isomerized to generate lactic acid [43]. With glucose as the substrate at 180 °C for 30 min (Figures S1–S4), the main products consist of lactic acid (76.0%), glucose (0.9%), fructose (1.6%), sorbitol (1.2%), xylitol (0.4%), levulinic acid (7.8%), HMF (4.0%) and formic acid (1.1%). All of the C3 products (1,3-dihydroxyacetone, glycerol aldehyde, and pyruvaldehyde) shown in Figure S6 were mostly converted to lactic acid in the presence of Sn-Beta in more than 92.0% yields. Surprisingly, when the reaction was carried out with fructose as the substrate, the yield of lactic acid (58.9%) was lower than that when glucose served as the feedstock, and more levulinic acid (10.6%) and formic acid (1.8%) were formed as the by-products (Figure S6), so the presence of Brønsted acid may accelerate the dehydration of fructose into HMF. In the retro-aldol condensation of fructose to C3, a competing reaction for its dehydration to HMF also occurs. By modulating the acidity of Beta zeolite, its Lewis acidity was increased while its Brønsted acidity was decreased, leading to a catalyst system dominated by its Lewis acidity, which favored the reaction. According to our and others’ previous studies [25,44], glucose (−1.58 eV) has more negative adsorption energy than fructose (−1.51 eV), which can be more easily adsorbed at the Sn active sites, thus controlling the rate of its isomerization and limiting the accumulation of fructose and inhibiting to the dehydration of fructose to HMF (Scheme 1).

## 3. Experimental

### 3.1. Chemicals and Materials

All chemicals were used as received from the commercial suppliers: glucose (AR, 99.0%), fructose (≥99%), formic acid (AR, ≥99%), SnCl_4_·5H_2_O (AR, ≥99.0%), 1,3-dihydroxyacetone (DHA) (≥98%), pyruvaldehyde (PAL) (40.0%), glyceraldehyde (GLY) (≥99.0%), the Beta zeolite with SiO_2_/Al_2_O_3_ = 100 and SiO_2_/Al_2_O_3_ = 25 were obtained from Shanghai Titan Technology Co., Ltd. (Shanghai, China). Methanol (HPLC), 5-hydroxymethyl furfural (≥99%), xylitol (≥98%), sorbitol (AR, ≥98%), and levulinic acid (AR, ≥99.0%) were purchased from Shanghai Aladdin Reagent Co., Ltd. (Shanghai, China). Lactic acid (AR, 90%), H_3_PO_4_ (GR, ≥85.0%), and NaH_2_PO_4_ (AR, ≥99.0%) were obtained from National Pharmaceutical Reagent Co., Ltd. (Shanghai, China).

### 3.2. Synthesis of Sn-Beta Catalyst

**Figure 4:**
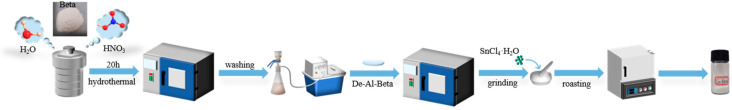
Preparation of the Sn-Beta catalyst.

Mix commercial Beta with 13 M HNO_3_ at a ratio of 1 g/20 mL, place in an oven, and hydrothermal at 100 °C for 20 h. Wash with deionized water until the pH of the supernatant is 7 to obtain dealuminated Beta and dry overnight at 100 °C. For solid-state ion exchange, add 0.06 g of SnCl_4_·H_2_O to 1 g of dealuminated Beta, grind thoroughly, and then roast 550 °C for 6 h (Figure 4).

### 3.3. Reactions and Liquid Phase Product Analysis

All experiments were conducted in a 100.0 mL reactor manufactured by Beijing Century Sen Lang Ltd., (China). The glucose and Sn-Beta catalyst with a certain mass ratio, along with 6.0 mL of water, were introduced into the reactor. This setup was maintained under a nitrogen atmosphere at a pressure of 2 MPa, while carefully controlling both the reaction time and the rate of reaction. Upon completion of the reaction, the reactor was swiftly immersed in cold water to facilitate rapid cooling. Subsequently, the reaction mixture underwent separation via centrifugation, and the resulting supernatant was filtered through 0.22 µm membranes to prepare it for quantitative analysis using high-performance liquid chromatography (HPLC).

The conversion of glucose was conducted utilizing an Agilent 1260 accompanied by a differential refractive index detector, employing an Aminex HPX-87H ion exclusion column with dimensions of 300 mm × 7.8 mm. The column was maintained at a temperature of 50 °C, and the injection volume was set at 60 μL. The mobile phase consisted of a 0.0125 mol/L aqueous solution of H_2_SO_4_, which was delivered at a flow rate of 0.60 mL/min. Subsequently, the liquid phase products were subjected to analysis via high-performance liquid chromatography (Shimadzu LC-20A), featuring an Ultimate TM AQ-C18 column measuring 100 mm × 4.6 mm. The analytical parameters were meticulously established as follows: the UV detector was calibrated to operate at a wavelength of 210 nm, the column temperature was maintained at 40 °C, and the injection volume was adjusted to 10 μL. The mobile phase utilized for this analysis was a 0.01 mol/L solution of H_3_PO_4_, with the pH = 2.5 adjusted by NaH_2_PO_4_, and the flow rate was set at 0.8 mL/min to ensure optimal separation and detection of the analytes.

### 3.4. Catalyst Characterization

The X-ray powder diffraction (XRD) patterns of the catalysts were acquired utilizing a Bruker D8 Advance X-ray diffractometer, which employed Cu Kα radiation. The scanning process was conducted within the 2θ range of 5° to 80° at a consistent rate of 2°/min.

To analyze the specific surface area, nitrogen adsorption was performed using the Brunauer–Emmett–Teller (BET) method with an ASAP 2420 instrument. Prior to testing, the sample underwent a degassing procedure at 300 °C for a duration exceeding 3 h, followed by nitrogen adsorption at −196 °C.

The UV–visible diffuse reflectance spectra were recorded across the wavelength range of 200–500 nm using the Shimadzu UV-2550 UV spectrophotometer, with barium sulfate (BaSO_4_) serving as the reference material.

Inductively Coupled Plasma Atomic Emission Spectrometry (ICP-AES) was executed with the Agilent 5110 Optical Emission Spectrometer (OES) to digest and filter a predetermined quantity of catalyst for the purpose of quantifying the content of Sn.

For the assessment of the valence state of tin, X-ray photoelectron spectroscopy (XPS) was conducted using the K-Alpha spectrometer from Thermo Fisher Scientific (U.S.) (Waltham, MA, USA). The morphology of the catalyst was evaluated through Scanning Electron Microscopy (SEM) analysis, which was performed with a Quanta FEG 250 at a test voltage of 10 kV. The sample was thoroughly dried and milled, after which a small amount was dispersed onto the conductive adhesive and subsequently gold-coated under vacuum conditions.

Pyridine adsorption Fourier-transform infrared spectroscopy (Py-IR) involved the drying of samples prior to testing. Approximately 10 mg of catalyst was weighed and pressed into uniform circular flakes with a diameter of 13 mm using a feeler tool. The sample canister was evacuated and subjected to treatment at 200 °C for 2 h, followed by cooling to room temperature for background spectrum measurement. Pyridine gas was then introduced to saturate the sample, and the temperature was elevated to 200 °C for 1 h to facilitate spectrum measurement. Subsequently, the temperature was increased to 350 °C for an additional hour to obtain infrared spectra of desaturation at both 200 °C and 350 °C.

The Ammonia-Temperature-Programmed Desorption (NH_3_-TPD) analysis was carried out utilizing a Micromeritics AutoChem II 2920 apparatus. In this procedure, 100 mg of the sample was positioned within a reaction tube and subjected to a heating protocol that increased the temperature from ambient conditions to 120 °C at a controlled rate of 10 °C per minute. Prior to the main analysis, a drying pretreatment was executed using a helium (He) gas flow at a rate of 30–50 mL/min for a duration of 1 h. Following this, the sample was allowed to cool to 50 °C, where it was then exposed to 10% ammonia (NH_3_) in a helium mixture, also at a flow rate of 30–50 mL/min, for an additional hour to ensure saturation of the sample. After saturation, the gas flow was reverted back to pure helium at the same flow rate for another hour to effectively purge any weakly adsorbed NH_3_ from the sample matrix. The desorption of the weakly physically adsorbed NH_3_ was subsequently achieved at a high temperature of 600 °C under a helium atmosphere, employing a temperature ramp of 10 °C/min. The desorbed gases were then analyzed and quantified using a thermal conductivity detector (TCD).

In addition, Total Organic Carbon (TOC) analysis was performed with an Analytik Jena multi-N/C 2100 instrument, which necessitated the dilution of liquid products within a concentration range of 0 mg/L to 100 mg/L. The results obtained from the TOC analysis demonstrated a carbon balance that was remarkably close to 100% at a temperature of 180 °C.

Furthermore, thermogravimetric analysis (TGA) was executed using a Netzsch STA449F5 instrument under either helium or air flow conditions. For this analysis, 10 mg of the sample was placed in a crucible and subjected to a heating profile that ranged from 40 °C to 900 °C, allowing for the observation and quantification of weight loss throughout the temperature increase.

## 4. Conclusions

In the present study, Sn-Beta catalysts with dual Lewis and Brønsted acid active sites were prepared by a simple post-synthesis method, and when using glucose as the substrate, a lactic acid yield of 76.0% was obtained at 180 °C after 30 min. A series of characterizations showed that the 2 wt% Sn is uniformly dispersed in the skeleton structure of the fully de-aluminized Beta zeolite; dominated Lewis acid sites were formed by modulating the acidity of the Beta zeolite, which inhibited that side reactions and was vital to the excellent catalytic performance. Meanwhile, glucose could be adsorbed more easily than fructose at the Sn active sites, limiting the accumulation of fructose, which inhibited fructose dehydration to HMF and promoted the production of lactic acid. We hope that this work can provide guidance for the industrial production of lactic acid extracted from biomass.

## Data Availability

The original contributions presented in this study are included in the article and Supplementary Materials.

## Supplementary Materials

The following supporting information can be downloaded at: https://www.mdpi.com/article/10.3390/molecules30071457/s1. Table S1. Tin content in the catalysts. Table S2. BET of Beta, deAl-Beta and Sn-Beta catalyst. Table S3. Py-IR analyses of Beta, deAl-Beta and Sn-Beta catalyst. Table S4. NH3-TPD analyses of Beta, deAl-Beta and Sn-Beta catalyst. Figure S1. Product distribution for glucose convention over Sn-Beta catalyst at different temperatures. Reaction conditions: 200.0 mg glucose, 20.0 mL deionized water, 200.0 mg Sn-Beta catalyst, 2 MPa N2, 30 min. Figure S2. Product distribution of glucose convention over Sn-Beta catalysts as a function of time. Reaction conditions: 180 °C, 200.0 mg glucose, 20.0 mL deionized water, 200.0 mg Sn-Beta catalyst, 2 MPa N2. Figure S3. Product distribution of glucose convention over Sn-Beta catalyst under different pressure. Reaction conditions: 180 °C, 30 min, 200.0 mg glucose, 20.0 mL deionized water, 200.0 mg Sn-Beta catalyst. Figure S4. Product distribution of glucose convention over Sn-Beta catalyst under different feedstock-to-catalyst ratios. Reaction conditions: 180 °C, 30 min, 20.0 mL deionized water, 2 MPa N2. Figure S5. (a) SEM and (b) TEM-mapping images of Sn-Beta. Figure S6. Product distributions and convention rates for the convention of different feedstocks over the Sn-Beta catalyst. Reaction conditions: 180 °C, 30 min, 200.0 mg feedstocks, 20.0 mL deionized water, 2 MPa N2, 300.0 mg Sn-Beta catalyst. Pyruvaldehyde (PAL); Glyceraldehyde (GLY); 1,3-dihydroxyacetone (DHA). Figure S7. Effect of the number of cycles on the product distribution. Reaction conditions: 180 °C, 200.0 mg glucose, 20.0 mL deionized water, 200.0 mg Sn-Beta catalyst, 2 MPa N2, 30 min. Figure S8. TG of fresh and used Sn-Beta. Figure S9. XRD patterns of fresh, used and regenerated Sn-Beta.
