# Supplementary material for: Efficient Conversion of Glucose into Lactic Acid over the Lewis Acidity Enhanced Sn-Beta Catalyst
Source: Molecules. 2025 Mar 25;30(7):1457. doi: 10.3390/molecules30071457 (PMC11990729; doi:10.3390/molecules30071457)
Supplement: Supplementary file 1 [file molecules-30-01457-s001.zip › molecules-3531361-supplementary.pdf]

Supporting Information

Efficient conversion of glucose into lactic acid over the Lewis acidity enhanced Sn-Beta catalyst

Fenfen Guo [1], Yuxuan Wang [1], Zhicheng Jiang [1], Youjing Tu [1], Ruikai Li [1], Xingyu Zhang [1], Aoyi Tang [1], Yuan Liang [1], Lishi Yan [2], Shenggang Li [3], Hu Luo [3]* Lingzhao Kong [1]*

[1] School of Environmental Science and Engineering, Suzhou University of Science and Technology, Suzhou, Jiangsu, 215009, P.R. China; 2213022037@post.usts.edu.cn (F.G.); 2213022044@post.usts.edu.cn (Y.W.); 2213022010@post.usts.edu.cn (Z.J.); 2313021012@post.usts.edu.cn (Y.T.); 2313021004@post.usts.edu.cn (R.L.); 2313022025@post.usts.edu.cn (X.Z.); 2313021008@post.usts.edu.cn (A.T.); liangyuan@mail.usts.edu.cn (Y.L.)

[2] School of Chemistry and Life Sciences, Suzhou University of Science and Technology, Suzhou, Jiangsu, 215009, P.R. China; yanls@usts.edu.cn (L.Y.)

[3] CAS Key Laboratory of Low-Carbon Conversion Science and Engineering, Shanghai Advanced Research In-stitute, Chinese Academy of Sciences, Shanghai 201210, P.R. China; , lisg@sari.ac.cn (S.L.).

[*] Correspondence: luoh@sari.ac.cn (H.L.), konglz@sari.ac.cn (L.K.).

Contents

1. Yield Calculations 1
2. Cycling Experiment 1
3. Results and Discussion 2

1. Yield Calculations

All product yields are calculated as carbon yields.

Yield = (moles of carbon in the products determined by HPLC) ÷ (moles of carbon in feedstock) × 100%.

2. Cycling Experiment

At the end of the catalytic reaction (0.2 g Sn-Beta catalyst, 20 ml water, 180 °C, 0.5 h), the catalyst was separated from the reaction solution by centrifugation, the solids were washed briefly several times with purified water and vacuum dried overnight at room temperature before the next run was performed.

3. Results and Discussion

Table S1. Tin content in the catalysts

Catalysts	Sn % (Theoretical loading)	Sn % (Actual loading)	Sn % (After reaction)
Beta	0	0	0
Sn-Beta	2	1.72	1.66

Table S2. BET of Beta, deAl-Beta and Sn-Beta catalyst.

Samples	$S_{BET}(m^2g^{-1})$	Micropore area ($m^2\ g^{-1}$)	Micropore volume ($mL\ g^{-1}$)	Pore width/ (nm)
Beta	642.93	514.71	0.20	6.14
deAl-Beta	546.03	413.31	0.17	4.55
Sn-Beta	635.74	493.58	0.19	4.99

Table S3. Py-IR analyses of Beta, deAl-Beta and Sn-Beta catalyst.

Samples	Temperature(°C)	Lewis site density (μmol g^{-1})	Brønsted site density (μmol g^{-1})	Total[1]	B/L[2]
Beta	200	41.55	7.47	49.03	0.18
	350	32.67	4.86	37.53	0.15
deAl-Beta	200	14.25	0.49	14.75	0.03
	350	0.25	0.92	1.17	3.66
Sn-Beta	200	73.9	2.13	76.03	0.03
	350	1.72	2.11	3.83	1.23

Total[1]: Total acid content in catalyst, The Sn loading is 2 wt% in all experiments.

B/L[2]: The acid sites density ratio of Brønsted acid: Lewis acid.

Table S4. NH_3-TPD analyses of Beta, deAl-Beta and Sn-Beta catalyst.

Samples	Acid Site Density (μmol g^{-1})		
	<200 °C	>450 °C	Total[1]
Beta	357.75	168.98	526.73
Sn-Beta	517.2	255.92	733.12

Total[1]: Total acid content in catalyst, The Sn loading is 2 wt% in all experiments.

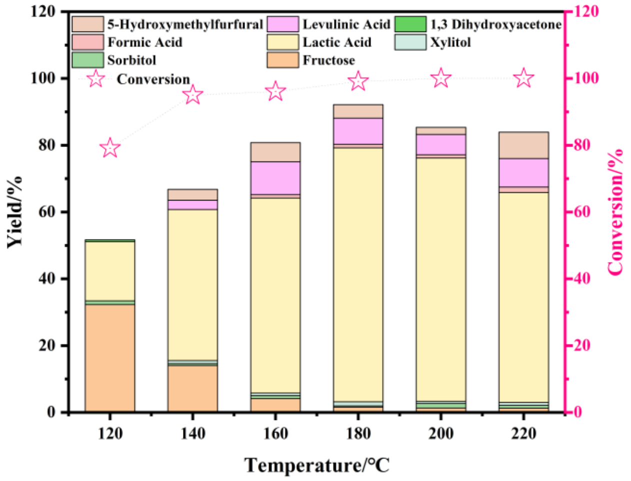

Figure S1. Product distribution for glucose convention over Sn-Beta catalyst at different temperatures. Reaction conditions: 200.0 mg glucose, 20.0 mL deionized water, 200.0 mg Sn-Beta catalyst, 2 MPa N_2,30 min.

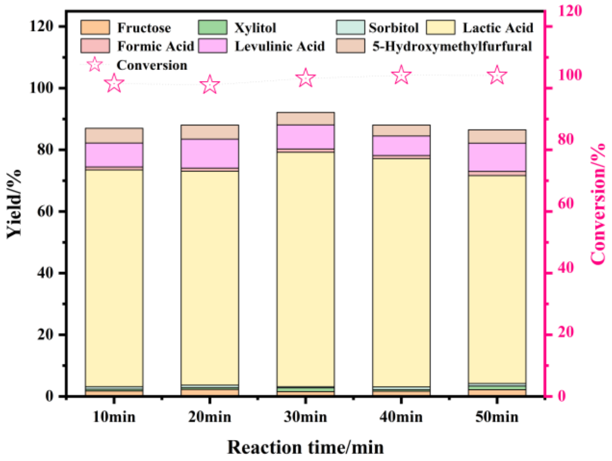

Figure S2. Product distribution of glucose convention over Sn-Beta catalysts as a function of time. Reaction conditions: 180 °C, 200.0 mg glucose, 20.0 mL deionized water, 200.0 mg Sn-Beta catalyst, 2 MPa N_2.

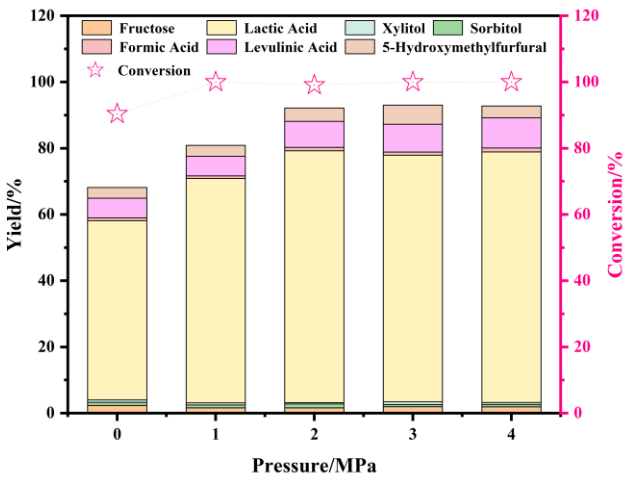

Figure S3. Product distribution of glucose convention over Sn-Beta catalyst under different pressure. Reaction conditions: 180 °C, 30 min, 200.0 mg glucose, 20.0 mL deionized water, 200.0 mg Sn-Beta catalyst.

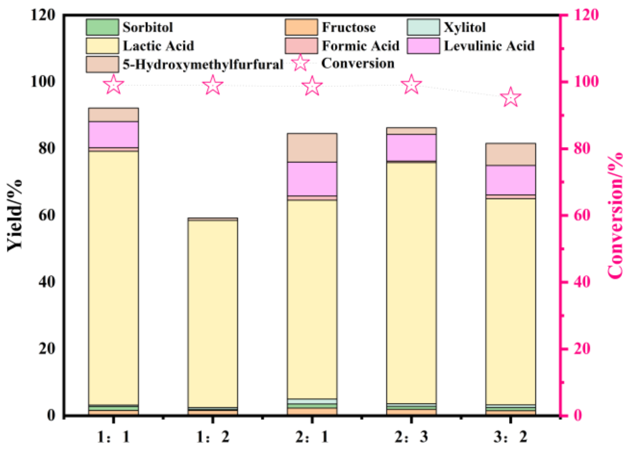

Figure S4. Product distribution of glucose convention over Sn-Beta catalyst under different feedstock-to-catalyst ratios. Reaction conditions: 180 °C, 30 min, 20.0 mL deionized water, 2 MPa N_2.

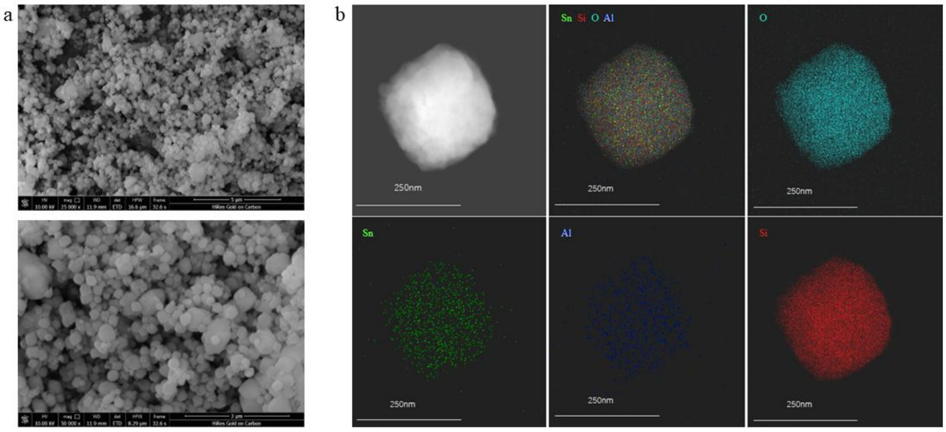

Figure S5. **(a)** SEM and **(b)**TEM-mapping images of Sn-Beta

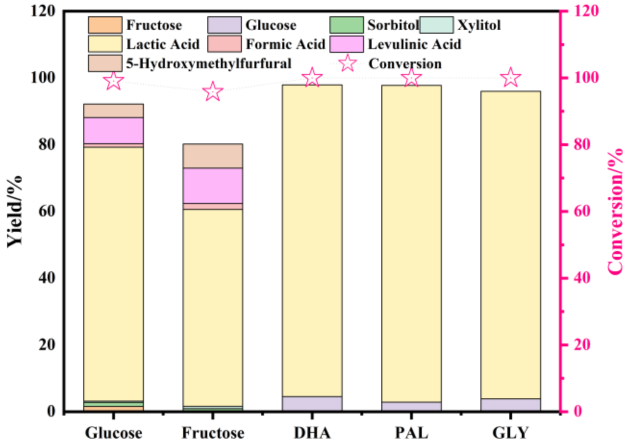

Figure S6. Product distributions and convention rates for the convention of different feedstocks over the Sn-Beta catalyst. Reaction conditions: 180 °C, 30 min, 200.0 mg feedstocks, 20.0 mL deionized water, 2 MPa N_2, 300.0 mg Sn-Beta catalyst. Pyruvaldehyde (PAL); Glyceraldehyde (GLY); 1,3-dihydroxyacetone (DHA).

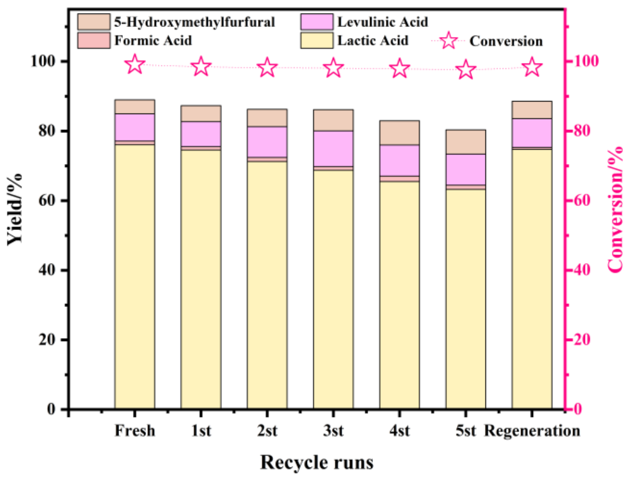

Figure S7. Effect of the number of cycles on the product distribution.Reaction conditions: 180 °C, 200.0 mg glucose, 20.0 mL deionized water, 200.0 mg Sn-Beta catalyst, 2 MPa N_2, 30 min.

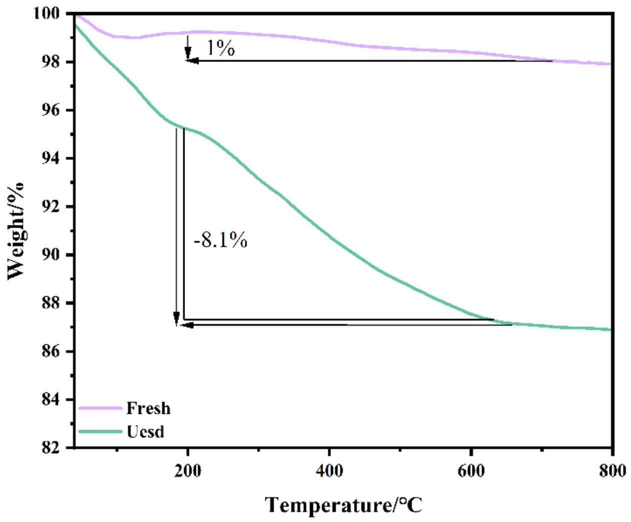

Figure S8. TG of fresh and used Sn-Beta.

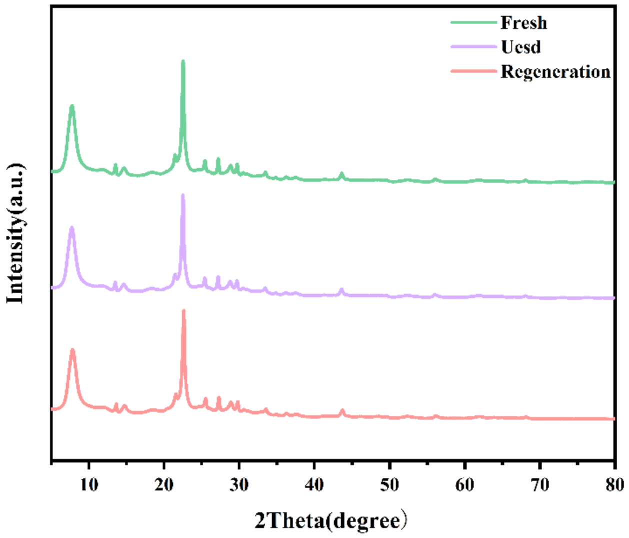

Figure S9. XRD patterns of fresh, used and regenerated Sn-Beta.
